# Mental health care-seeking and barriers: a cross-sectional study of an urban Latinx community

**DOI:** 10.1186/s12889-024-20533-6

**Published:** 2024-11-08

**Authors:** Jennifer A. Newberry, Michelle A. Gimenez, Fatma Gunturkun, Erica Villa, Maritza Maldonado, Dilza Gonzalez, Gabriel Garcia, Patricia Rodriguez Espinosa, Haley Hedlin, Debra Kaysen

**Affiliations:** 1 Department of Emergency Medicine, Stanford University School of Medicine, Stanford, CA USA; 2 Department of Medicine, Stanford University School of Medicine, Stanford, CA USA; 3 Next Door Solutions to Domestic Violence, San Jose, CA USA; 4 Amigos de Guadalupe, San Jose, CA USA; 5 SOMOS Mayfair, San Jose, CA USA; 6 Department of Epidemiology and Population Health, Stanford University School of Medicine, Stanford, CA US; 7 Department of Psychiatry and Behavioral Sciences, Stanford University School of Medicine, Stanford, CA USA

**Keywords:** Mental health, Help-seeking, Latinx

## Abstract

**Background:**

The Latinx community faces an increasing amount of mental health challenges and disparities in care. While the contributing factors are complex, there are likely potential barriers related to connecting with mental health support and accessing care that can be addressed.

**Methods:**

To investigate barriers in connecting to mental health care, we conducted a cross-sectional survey of mental health service use and barriers in an urban community with a primarily Hispanic/Latinx ethnicity using a modified random walk approach for door-to-door data collection with a two-cluster sampling frame. Survey included questions on socio-demographic, mental health status, desire and attempt to seek care, and the Barriers to Access to Care Evaluation. Shapley additive explanation (SHAP) identified impactful barriers and demographic characteristics. Our primary outcome was the number of respondents who saw a professional in the past 12 months and the key determinants that enabled their successful connection. Secondary outcomes were people with poor mental health who had wanted or tried to seek any source of mental health support.

**Results:**

Of the 1004 respondents enrolled, 70.5% were foreign born; 63.4% were women. In the past 12 months, 23.8% of respondents wanted to connect with mental health care; 15.5% tried to connect, and only 11.7% successfully connected to mental health services. The two most cited barriers had the highest SHAP values: concerns about treatments available (65%) and financial costs (62.7%). Additional barriers with high SHAP values: being seen as weak and having no one to help them find care. Of demographic characteristics, age had the highest SHAP values.

**Conclusion:**

In a community with a high density of Latinx immigrants, just under half of respondents wanting mental health care successfully connected. Perceived informational, financial, and stigma-related barriers impacted the likelihood to connect with mental health care. These factors should be considered when designing programs and interventions to improve mental health care access and services in the Latinx community.

**Supplementary Information:**

The online version contains supplementary material available at 10.1186/s12889-024-20533-6.

## Background

The Latinx community, comprised of almost 60 million people in the U.S [1]. , faces a widening mental health treatment gap caused by structural determinants of health [2]. Among Hispanic people with any mental illness (6.9 million), almost 70% will not receive needed treatment [3]. Recognized structural disparities include high levels of poverty [4], high levels of uninsured and underinsured [4], low levels of educational attainment and English proficiency [5], and stigma surrounding mental health [6]. In addition, not perceiving a need for mental health care is one of the most common reasons for not seeking treatment [7] and is particularly common in the Latinx community, contributing to disparities in mental health care connection and treatment [2]. 

Beyond the perceived need for mental health care, studies have shown minority populations and marginalized communities in the U.S. may experience more barriers to accessing care, including sexual and gender minorities with disabilities [8], aging and geriatric populations [9], and Muslim immigrants [10]. Specifically, within the Hispanic and Latinx community, barriers to receiving care have been associated with English language proficiency levels, immigration status, traditions related to culture and gender norms, and differing social needs and religiosity [11, 12]. While stigma around mental health and connecting to mental health care are common phenomena present in many Latinx communities [6], only a few studies exist focused on examining mental health help-seeking beliefs in Latinx communities [2]. Existing studies that assess perceived barriers to accessing professional care for mental illness and beliefs, including stigma, availability of service, and accessibility, are hampered by small sample sizes [13, 14]. In contrast, the Center for Disease Control and Prevention’s (CDC) Behavioral Risk Factor Surveillance System (BRFSS) assesses self-rated mental health status and impacts of mental health on daily life from a nationally representative sample of U.S. residents across all 50 states annually, but does not collect data on barriers to mental health care [15]. 

To better understand barriers to mental health care connection in the Latinx community, we conducted a cross-sectional survey about mental health help-seeking patterns and barriers in our local community. As survey collection faces its own challenges among the Latinx community, including language, trust, and reduced participation [16], we chose to take a community-engaged approach to research design and implementation, working closely with community partners and Spanish-speaking community health workers, known as *promotores de salud* (hereafter, promotores), as the primary data collectors, as they bring with them established trust and deep knowledge of their local communities. By identifying barriers to help-seeking and connection to mental health care, the results of this survey will help to inform more targeted mental health care interventions to improve access and utilization in the Latinx community.

## Methods

### Study design

We conducted a cross-sectional survey of mental health care use and barriers in a single urban community in Northern California from March through May 2023. Inclusion criteria for participation were: (1) self-identify as Latinx or Hispanic, (2) be 18 years and older, (3) be able to understand and speak English or Spanish, and (4) have a primary residence in zip codes 95,116, 95,122, or 95,127. These zip codes were chosen because they represent East San José, an urban area, with 57–62% Hispanic people [1], compared to 39% of California’s overall Hispanic/Latinx population and 18% nationally. In addition, 41–52% of people in East San José are foreign born (CA: 27%); 64–75% have a high school degree or higher (CA: 84%); 6–9% are uninsured (CA: 7%); and 34–42% are covered by Medi-Cal (CA: 38%) [1].

Persons were unable to participate if: (1) another person in the household had already responded to the survey, (2) they were unable to provide verbal consent, or (3) they could not complete the survey in either English or Spanish. We sought to sample 1,000 distinct households. This study was approved by our institutional review board. All community partners contributed to, reviewed, and provided feedback on study protocols.

### Data collection

Each data collection field team consisted of at least one bilingual research assistant and one to two promotores recruited from three separate San José community-based organizations. All promotores, who were monolingual Spanish or bilingual English/Spanish speakers, were trained on study protocols.

We randomly sampled households using a modified random walk approach with a two-cluster sampling frame [17, 18]. The primary sampling unit was census block groups, and the secondary sampling unit was households within those census block groups. Census block groups within a given zip code were chosen using a random number generator and each week a data collection field team was assigned a census block group within a zip code. We removed census block groups with less than 50 homes, partially within non-target zip codes, or with a density of Hispanic homes < 20% per the most recent census. Selection of the individual households approached within a given census block group primarily relied on the contextual knowledge of the promotores (e.g. likelihood of being home, safety). Data collection occurred six days a week (Tuesday-Sunday) during daytime and early evening hours.

### Measures

The survey (available upon request) included questions on socio-demographics, insurance, nativity, and years in the U.S. We also included age, race, ethnicity, marital status, and gender. While all respondents identified as Hispanic or Latinx, we also asked if they identified with any additional specific communities, such as Mexican, Colombian, Salvadoran, Honduran, or other. The listed subgroups were chosen as they are the most populous in the area.

The survey also included questions on perceived discrimination [19], self-reported mental health status, and study-specific questions on the desire and attempt(s) to connect to mental health care. We asked about mental health both as an overall self-assessment with a 5-point Likert scale and as the number of poor mental health days experienced in the past 30 days. To understand prior mental health care help-seeking experiences, we asked respondents whether they had wanted to connect to care, tried to connect to care, or had successfully connected to care for mental health. We included two sets of study-specific questions on the mental health care help-seeking continuum, split by the type of support: (1) mental health specialists such as counselors, therapists, psychiatrists, or psychologists; (2) lay support such as religious leaders, traditional healers, herbalists, pharmacists, community elders, support groups, or other (not inclusive of informal support such as family or friends).

We also administered the Barriers to Access to Care Evaluation (BACE-3) instrument [20], using both an English version and a Spanish version [21]. The Spanish version was piloted with our team of promotores and minor adaptions were made. Each item is scored using a 4-point Likert scale by how much the barrier has ‘stopped, delayed, or discouraged’ the respondent from getting or continuing with care, ranging from 0 (‘not at all’) to 3 (‘a lot’). In this scale, higher scores are indicative of a greater degree of the barrier. The following prompt was added for those who had not previously connected to care: “We are interested in hearing more about potential barriers you think you would be likely to encounter when trying to connect with professional care.”

### Data analysis

Our primary outcome was the number of respondents who saw a professional in the past 12 months and the key determinants that enabled their successful connection. Secondary outcomes were people with poor mental health who had wanted or tried to seek any source of mental health support. We summarized respondent characteristics across the mental health care help-seeking continuum using median and interquartile range (IQR) for continuous variables, and frequencies and percentages for categorical variables, respectively. We report the means and standard deviations (SD) for the overall scores and for each item of the BACE-3 instrument, as well as the percentage of respondents who experienced the barrier to any degree or to a major degree (defined as ‘a lot’).

We created a decision tree in order to explore subgroups and identify the most important barriers associated with access to care. A decision tree was generated using a LightGBM model [22]. To mitigate the potential risk of overfitting and optimize the hyperparameters of our decision tree, we employed cross-validation to provide robust decision trees. Ultimately, we fit a single decision tree to describe subgroups that are more likely to receive or not receive care, rather than focusing on prediction. The rules, as well as the support and confidence levels of these rules, were derived from the tree. Here, ‘support (S)’ is the percentage of respondents the rule covers, while ‘confidence (C)’ indicates the percentage of those respondents in the majority for that rule. After building the decision tree model, we analyzed the Shapley additive explanation (SHAP) values to understand the feature value or importance, as well as the association between the variables and the outcome [23]. In short, the SHAP was used to identify the main barriers for connecting to care and to generate hypotheses for future work.

Statistical analyses were performed using R (version 4.2.2) and Python (version 3.7). This study adhered to the Strengthening the Reporting of Observational Studies in Epidemiology (STROBE) reporting guidelines for cross-sectional studies [24]. 

## Results

Of the 8241 households we approached, our contact rate was 37.6% (*N* = 3099) and our response rate was 32.4% (*N* = 1004). A STROBE diagram and map of the census block groups sampled can be found in the appendix. Most people did not answer the door (*N* = 5144). Of the households where someone was home, 1575 (50.8%) were not available or interested in hearing about the survey, 518 (16.7%) were not eligible primarily due to speaking a language other than English or Spanish, and only one person did not consent who was otherwise eligible.

Over 12 weeks, we enrolled 1004 respondents who were 63.5% women and had a median age of 45 (IQR 34–58) (Table 1). Respondents primarily chose to take the survey in Spanish, 76.8% (*N* = 771), and 70.1% (*N* = 704) were born outside of the United States.

**Table 1:** Respondent Demographics

	Total *N* (%) *	Poor Mental Health *N* (%) **	Wanted to Connect to Care *N* (%) **	Tried to Connect to Care *N* (%) **	Connected to Care *N* (%) **
**All**	1004	216 (21.5%)	240 (24.0%)	157 (15.6%)	118 (11.8%)
**Age group**					
Median (IQR)	45 (34–58)	48 (31.5–59)	39 (28–51)	38 (30–50)	38 (27–49.75)
<=25	121 (12.1%)	37/121 (30.6%)	48/121 (39.7%)	31/121 (25.6%)	25/121 (20.7%)
25–50	495 (49.3%)	87/495 (17.6%)	128/495 (25.9%)	89/495 (18.0%)	66/495 (13.3%)
> 50	387 (38.5%)	91/387 (23.5%)	64/387 (16.5%)	37/387 (9.6%)	27/387 (7.0%)
Missing	1 (0.1%)	1/1 (100.0%)	0/1 (0.0%)	0/1 (0.0%)	0/1 (0.0%)
**Gender**					
Male	355 (35.4%)	67/355 (18.9%)	73/355 (20.6%)	43/355 (12.1%)	33/355 (9.3%)
Female	638 (63.5%)	146/638 (22.9%)	161/638 (25.2%)	110/638 (17.2%)	81/638 (12.7%)
Prefer not to answer	6 (0.6%)	3/6 (50.0%)	4/6 (66.7%)	2/6 (33.3%)	2/6 (33.3%)
**Race**					
White	196 (19.5%)	42/196 (21.4%)	53/196 (27.0%)	30/196 (15.3%)	24/196 (12.2%)
Black or African American	18 (1.8%)	3/18 (16.7%)	7/18 (38.9%)	6/18 (33.3%)	6/18 (33.3%)
American Indian or Alaskan Native	60 (6.0%)	21/60 (35.0%)	18/60 (30.0%)	13/60 (21.7%)	10/60 (16.7%)
Asian	11 (1.1%)	2/11 (18.2%)	4/11 (36.4%)	2/11 (18.2%)	2/11 (18.2%)
Other	648 (64.5%)	133/648 (20.5%)	144/648 (22.2%)	96/648 (14.8%)	71/648 (11.0%)
Two or more races	36 (3.6%)	7/36 (19.4%)	9/36 (25.0%)	6/36 (16.7%)	6/36 (16.7%)
**Ethnicity subgroup**					
Mexican, Mexican American, Chicano	878 (87.5%)	198/878 (22.6%)	206/878 (23.5%)	135/878 (15.4%)	104/878 (11.8%)
Other Latinx or Hispanic	131 (13.0%)	16/131 (12.2%)	35/131 (26.7%)	25/131 (19.1%)	17/131 (13.0%)
**Insurance type**					
Private	354 (35.3%)	64/354 (18.1%)	82/354 (23.2%)	49/354 (13.8%)	41/354 (11.6%)
Public	519 (51.7%)	122/519 (23.5%)	141/519 (27.2%)	98/519 (18.9%)	72/519 (13.9%)
Not covered	126 (12.5%)	25/126 (19.8%)	18/126 (14.3%)	12/126 (9.5%)	7/126 (5.6%)
**Marital status**					
Married	454 (45.2%)	76/454 (16.7%)	78/454 (17.2%)	47/454 (10.4%)	34/454 (7.5%)
Divorced/Separated	129 (12.9%)	10/129 (7.8%)	37/129 (28.7%)	25/129 (19.4%)	19/129 (14.7%)
Widowed	53 (5.3%)	14/53 (26.4%)	8/53 (15.1%)	5/53 (9.4%)	3/53 (5.7%)
Never married	208 (20.7%)	56/208 (26.9%)	74/208 (35.6%)	48/208 (23.1%)	42/208 (20.2%)
A member of an unmarried couple	134 (13.3%)	32/134 (23.9%)	37/134 (27.6%)	28/134 (20.9%)	17/134 (12.7%)
Missing	26 (2.6%)	6/26 (23.1%)	6/26 (23.1%)	4/26 (15.4%)	3/26 (11.5%)
**Number of children**					
None	503 (50.1%)	123/503 (24.5%)	123/503 (24.5%)	81/503 (16.1%)	67/503 (13.3%)
Kids in house	435 (43.3%)	80/435 (18.4%)	107/435 (24.6%)	72/435 (16.6%)	48/435 (11.0%)
Kids out of house	61 (6.1%)	12/61 (19.7%)	9/61 (14.8%)	3/61 (4.9%)	2/61 (3.3%)
Missing	5 (0.5%)	1/5 (20.0%)	1/5 (20.0%)	1/5 (20.0%)	1/5 (20.0%)
**Education**					
Never attended	83 (8.3%)	27/83 (32.5%)	14/83 (16.9%)	8/83 (9.6%)	5/83 (6.0%)
12th grade or less, no diploma	370 (36.9%)	88/370 (23.8%)	71/370 (19.2%)	49/370 (13.2%)	32/370 (8.6%)
High school graduate	295 (29.4%)	55/295 (18.6%)	70/295 (23.7%)	43/295 (14.6%)	36/295 (12.2%)
Some college, no degree	121 (12.1%)	25/121 (20.7%)	45/121 (37.2%)	28/121 (23.1%)	23/121 (19.0%)
Other degree or certificate	52 (5.2%)	10/52 (19.2%)	12/52 (23.1%)	9/52 (17.3%)	4/52 (7.7%)
Bachelor or Graduate degree	77 (7.7%)	10/77 (13.0%)	28/77 (36.4%)	20/77 (26.0%)	18/77 (23.4%)
Missing	6 (0.6%)	0/6 (0.0%)	0/6 (0.0%)	0/6 (0.0%)	0/6 (0.0%)
**Education in US**					
Completed in US	415 (41.3%)	97/415 (23.4%)	140/415 (33.7%)	92/415 (22.2%)	77/415 (18.6%)
Not completed in US	585 (58.3%)	119/585 (20.3%)	100/585 (17.1%)	65/585 (11.1%)	41/585 (7.0%)
Missing	4 (0.4%)	0/4 (0.0%)	0/4 (0.0%)	0/4 (0.0%)	0/4 (0.0%)
**Nativity**					
Born in US	296 (29.5%)	78/296 (26.4%)	109/296 (36.8%)	70/296 (23.6%)	60/296 (20.3%)
Foreign born	704 (70.1%)	138/704 (19.6%)	131/704 (18.6%)	87/704 (12.4%)	58/704 (8.2%)
Missing	4 (0.4%)	0/4 (0.0%)	0/4 (0.0%)	0/4 (0.0%)	0/4 (0.0%)
**Immigration generation**					
1st generation	627 (62.5%)	125/627 (19.9%)	111/627 (17.7%)	70/627 (11.2%)	45/627 (7.2%)
1.5 generation	63 (6.3%)	9/63 (14.3%)	15/63 (23.8%)	13/63 (20.6%)	12/63 (19.0%)
Other	314 (31.3%)	82/314 (26.1%)	114/314 (36.3%)	74/314 (23.6%)	61/314 (19.4%)
**Language preference**					
Spanish	771 (76.8%)	157/771 (20.4%)	141/771 (18.3%)	96/771 (12.5%)	66/771 (8.6%)
English	233 (23.2%)	59/233 (25.3%)	99/233 (42.5%)	61/233 (26.2%)	52/233 (22.3%)
English language proficiency***					
Very good	89 (11.5%)	19/89 (21.3%)	17/89 (19.1%)	16/89 (18.0%)	13/89 (14.6%)
Good	132 (17.1%)	20/132 (15.2%)	24/132 (18.2%)	17/132 (12.9%)	15/132 (11.4%)
Not very well	390 (50.6%)	77/390 (19.7%)	79/390 (20.3%)	51/390 (13.1%)	31/390 (7.9%)
Not at all	114 (14.8%)	34/114 (29.8%)	16/114 (14.0%)	10/114 (8.8%)	5/114 (4.4%)
Don’t know	29 (8%)	3/29 (10.3%)	3/29 (10.3%)	1/29 (3.4%)	1/29 (3.4%)
Prefer not to answer	10 (3%)	2/10 (20.0%)	0/10 (0.0%)	0/10 (0.0%)	0/10 (0.0%)
Does not apply	1 (1%)	0/1 (0.0%)	0/1 (0.0%)	0/1 (0.0%)	0/1 (0.0%)
Missing	6 (8%)	2/6 (33.3%)	2/6 (33.3%)	1/6 (16.7%)	1/6 (16.7%)
**Frequency of experiencing unfair treatment by people in helping jobs because of the race/ethnic group**					
Never	736 (73.3%)	143/736 (19.4%)	145/736 (19.7%)	96/736 (13.0%)	70/736 (9.5%)
Once in a while	122 (12.2%)	31/122 (25.4%)	50/122 (41.0%)	35/122 (28.7%)	28/122 (23.0%)
Sometimes	105 (10.5%)	32/105 (30.5%)	33/105 (31.4%)	18/105 (17.1%)	15/105 (14.3%)
A lot	24 (2.4%)	7/24 (29.2%)	10/24 (41.7%)	7/24 (29.2%)	4/24 (16.7%)
Missing	17 (1.7%)	3/17 (17.6%)	2/17 (11.8%)	1/17 (5.9%)	1/17 (5.9%)

Data presented as *N* (%), unless otherwise noted

*% in this column are column precents using 1004 as the denominator

**% are row precents using the denominator in the Total column

*** Only asked to participants taking the survey in Spanish

With respect to our primary outcome, 11.8% successfully connected to care (*N* = 118). 788 respondents (78.5%) reported good mental health and 216 (21.5%) reported poor mental health. Of all respondents, 24% of respondents (*N* = 240) wanted to connect to care, and 15.6% tried to connect to care (*N* = 157). For our secondary outcome, of the 216 respondents reporting poor mental health, and 98 (45.3%) wanted to connect to mental health care. Ultimately, 50 of the respondents reporting poor mental health connected to mental health care (23.1%), compared to 68 of respondents reporting good mental health (8.6%). 18.4% (*N* = 185) of all respondents reported that they wanted to see a lay provider for mental health care, such as a traditional healer, herbalist, priest, or pharmacist, in the past 12 months (see appendix). Ultimately 10.1% of respondents (*N* = 101) were successful in getting care. Of note, amongst all people who wanted to connect to help (*N* = 185), 47% of respondents (*N* = 87) reported having good mental health.

Only 2.3% of respondents (*N* = 23) overall had ever sought care in an emergency department for mental health support. Notably, respondents using the ED were more likely to be Spanish speaking compared to respondents who sought mental health care outside of the ED (82.6% vs. 48.9%, *p* = 0.01). More of the respondents who sought care in the ED were foreign born (60.9% vs. 46.8%, *p* = 0.33) and publicly insured (69.6% v 61.1%, *p* = 0.09), but the association was not statistically significant.

Nearly all respondents (95.2%, *N* = 956) felt that they would experience at least one barrier to connecting with professional mental health care. Interestingly, for 20 of the possible 30 barriers, at least 1/3 of respondents reported them as a possible barrier (Table 2). The most cited individual barriers were ‘concerns about the treatment available’ (65.0%), ‘not being able to afford the financial costs of care’ (62.9%), and ‘wanting to solve the problem on my own’ (62.7%). Compared to respondents who did not want to connect to care, those who did want to connect had higher overall BACE scores (mean:0.54 [SD:0.44] vs. 0.84 [0.50], *p* < 0.001). For respondents who wanted to connect to care but did not connect compared to those that did successfully connect to care, there was no difference in overall BACE scores (0.81 [0.46] vs. 0.86 [0.54], *p* < 0.46).

**Table 2:** Overall BACE score and item scores

ITEMS	Mean (SD)	Barrier to any degree	Major barrier
**Overall BACE Score**	**0.61 (0.47)**		
**Stigma-related barrier items**	0.47 (0.57)		
Feeling embarrassed or ashamed.	0.35 (0.72)	23.9%	7.5%
Concern that it might harm my chances when applying for jobs.	0.56 (0.91)	33.5%	15.1%
Concern that I might be seen as weak for having a mental health problem.	0.54 (0.88)	33.6%	13.7%
Concern about what my family might think, say, do or feel.	0.54 (0.88)	33.8%	14.0%
Not wanting a mental health problem to be on my medical records.	0.58 (0.91)	35.9%	15.0%
Concern that I might be seen as ‘crazy’.	0.57 (0.96)	31.6%	16.2%
Concern that people I know might find out.	0.31 (0.70)	20.3%	6.8%
Concern that people might not take me seriously if they found out I was having professional care.	0.43 (0.79)	28.9%	9.7%
Concern about what people at work might think, say or do.	0.32 (0.71)	21.9%	7.2%
Concern about what my friends might think, say or do.	0.27 (0.63)	18.7%	5.6%
Concern that I might be seen as a bad parent.	0.47 (0.88)	26.8%	13.9%
Concern that my children may be taken into care or that I may lose access or custody without my agreement.	1.13 (1.30)	48.0%	38.4%
**Attitudinal barrier items**	0.71 (0.47)		
Dislike of talking about my feelings, emotions, or thoughts.	0.56 (0.82)	39.2%	11.6%
Concerns about the treatments available (e.g. medication side effects).	1.13 (1.07)	65.0%	32.1%
Wanting to solve the problem on my own.	1.12 (1.08)	62.6%	33.5%
Thinking that professional care probably would not help.	0.51 (0.76)	38.0%	9.7%
Fear of being put in hospital against my will.	0.79 (1.14)	38.8%	24.4%
Thinking the problem would get better by itself.	0.71 (0.89)	48.4%	16.4%
Having had previous bad experiences with professional care for mental health.	0.44 (0.81)	28.3%	10.8%
Thinking I did not have a problem.	0.62 (0.85)	43.2%	12.4%
Preferring to get help from family or friends.	0.78 (0.90)	53.2%	17.0%
Preferring to get alternative forms of care.	0.49 (0.81)	34.3%	10.2%
**Instrumental barrier items**	0.68 (0.59)		
Not being able to afford the financial costs involved.	1.21 (1.15)	62.7%	37.6%
Being too unwell to ask for help.	0.57 (0.86)	38.1%	12.6%
Difficulty taking time off work.	0.52 (0.87)	32.5%	13.0%
Being unsure where to go to get professional care.	0.70 (1.00)	40.8%	18.0%
Problems with transport or travelling to appointments.	0.33 (0.74)	21.2%	8.2%
Having no one who could help me get professional care.	0.70 (0.93)	44.4%	19.0%
Professionals from my own ethnic or cultural group not being available.	0.78 (0.98)	48.0%	21.0%
Having problems with childcare while I receive professional care.	0.64 (0.96)	37.7%	17.7%

**Fig 1:**
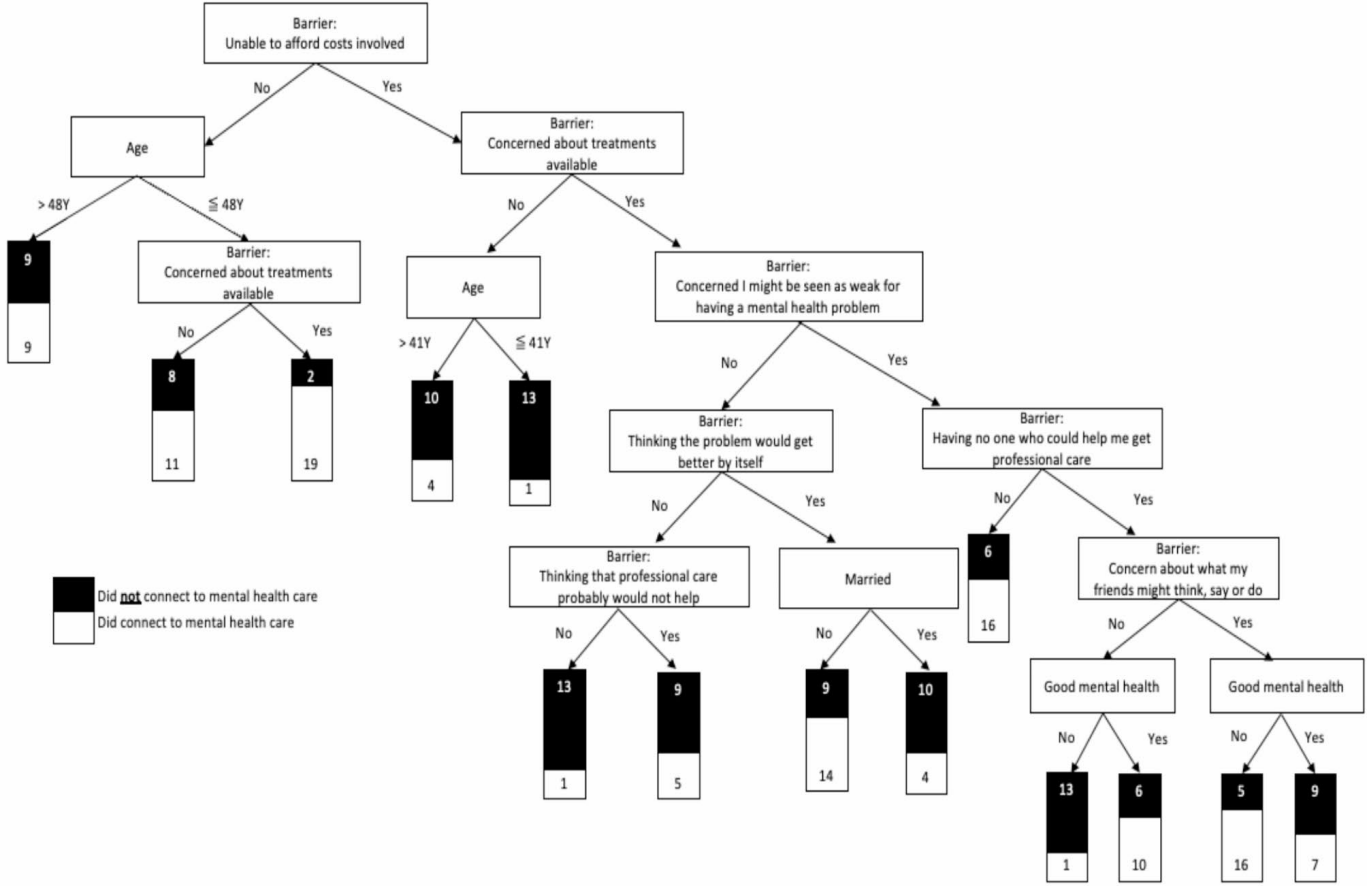
Decision Tree of LightGBM Model

The decision tree diagram of the LightGBM algorithm is illustrated in Fig 1. In creating the decision tree, we removed race because 64.6% of respondents were ‘other’, which upon free text review was primarily a restatement of Latinx. This is consistent with literature on the racialization of Latinx as a census category [5]. The algorithm generated a set of fourteen rules based on ten distinct features. The rules extracted from the decision tree indicate that respondents under the age of 48, with no affordability barrier but with concerns about available treatments, have an increased likelihood of connecting to mental health care (support (S):8.8%, confidence (C):90.5%). Conversely, the likelihood of connecting to mental health care was lower in respondents encountering barriers related to cost and being under the age of 41, but having no concerns about treatments available (S:5.8%, C:92.9%). Likewise, respondents encountering cost and treatment barriers, had no concerns about being seen as weak, who did not think the problem would get better, and thought that professional care would help, were associated with a decreased likelihood of connecting to care (S:5.8%, C:92.9%). As a sensitivity analysis, we repeated this approach using different ranges for parameters and numbers of splits in the cross-validation to observe how the most important barriers to receiving care varied across parameter settings. We found that the most important barriers remained consistent, confirming the robustness of our findings (see appendix).

**Fig 2:**
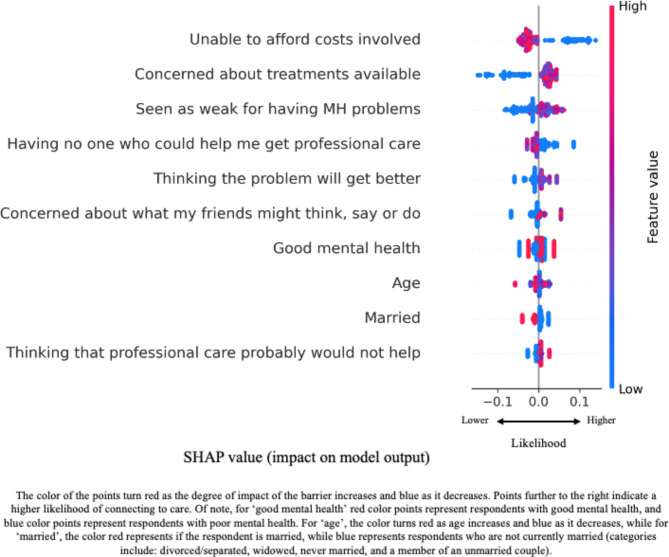
Summary Plot of Shapley Additive Explanation (SHAP) Values for Features Impacting Likelihood to Connect

Figure 2 shows the summary of the SHAP values for the most impactful factors noted using the LightGBM algorithm. Each point indicates one respondent. Features are sorted based on their importance for our primary outcome, with the top features being the most important. As depicted in the figure, the probability of individuals connecting to care decreases when the barrier associated with affordability concern increases (0–3, i.e., ‘not at all’ to ‘a lot’). Alternatively, for example, the barrier related to concerns about the treatments that are available increases the likelihood of connecting to care.

## Discussion

Using door-to-door data collection with a large sample size, we found that almost a quarter of this Latinx community wanted to connect to mental health care. However, only a small proportion of those who wanted to connect to mental health care actually attempted to seek help, and an even smaller proportion was successful in connecting with mental health care. The biggest gap between having poor mental health and connecting to mental health care was moving from wanting mental health care to trying to seek help. Further, this community perceived and experienced many barriers to getting mental health care. The two most impactful barriers to connecting to care were also the most common: being unable to afford treatment and concerns about available treatment.

Three of the most impactful barriers for connecting to care– ‘wanting to solve the problem on my own’, ‘concern that I might be seen as weak for having a mental health problem’, and  ‘concern about what my friends may think’ – require further investigation and contextualization within Latinx culture and align with existing barriers noted in Latinx populations in other regions of the U.S [25]. Specifically, these phenomena may reflect traditional roles and cultural beliefs held within this community, such as *machismo*,* caballerismo*, and *marianismo.* While certain aspects of *machismo* and *marianismo* have been associated with decreased help-seeking behaviors [26, 27], *caballerismo* has been associated with health promoting behaviors [28]. The data is mixed regarding efficacy of mental health stigma interventions among Latinx individuals, although the field is hampered by small pilot studies with low methodological rigor [29]. A systematic review of mental health literacy and stigma interventions for the Latinx community in the U.S., found that while studies used a variety of intervention designs (e.g., disseminating printed materials, hosting group/individual sessions), the strategies that proved most successful for increasing mental health literacy were the interventions that created and delivered content that engaged the participants in culturally and linguistically relevant ways [30]. This is consistent with our study, which highlights barriers with potential cultural roots. A better understanding of these cultural norms and their relation specifically to mental health care seeking will be critical to designing successful stigma-reduction interventions that move beyond basic accessibility factors, such as language.

While the financial cost of care is the leading reason for those with serious mental illness to not receive care, the prevalence of cost as a barrier is much higher in our study population than prior estimates (62.7% vs. 37.7%) [31]. Only 12.6% of our population was uninsured, which aligns with California’s state average of uninsured Latinx individuals [32]. Even with health insurance, the cost of mental health care remains significant: privately insured individuals are over five times as likely to have to pay out-of-network rates for behavioral health services as compared to medical or surgical services, despite federal and state laws requiring parity in coverage between mental and physical health services [33]. Given that our study shows financial costs are an impactful barrier to connecting with mental health care, future research should investigate the impact of the recent expansion of Medicaid in California to low-income adults regardless of immigration status. As prior Medicaid expansions have shown, we anticipate this will increase utilization of mental health care [34]. 

Of note, the most prevalent *major* barrier was the concern that one’s children may be taken into care or that they would otherwise lose custody. This barrier – reported by over one-third of respondents – may have reflected a particular fear experienced within this community which includes a high number of immigrants. This fear may be rooted in trends seen in the child welfare system: Latinx children are overrepresented in the foster system in some states; [35] when removed, Latinx children are taken from their homes more swiftly; [36] and once removed Latinx children spend more time away from their families [37]. Thus, our study highlights that immigration-related fears and fears regarding CPS contact may deter individuals, particularly those with children [38], from seeking mental health care. Future research needs to explore how efforts to improve coordination with the child welfare system may have ancillary benefits in improving connection with mental health services. Additionally, immigration and rights-based fears that act as barriers to mental health care may be addressable by medical-legal partnerships which engage a closer collaboration between health care providers and lawyers and have precedence in the immigrant community [39]. 

Fewer first-generation immigrants compared to second and even 1.5-generation immigrants wanted to or eventually connected with mental health care. Similarly, those who preferred to speak Spanish and those with poorer English proficiency were less likely to connect with mental health care. Studies have noted that Latinx individuals are more likely than non-Latinx White individuals to report psychological distress [40], and the within group differences seen in our study are important to show the heterogeneity within the Latinx community. However, these demographics did not emerge as impactful factors when barriers and additional demographics were taken into consideration using a machine learning approach as we did with LightGBM.

### Limitations

This study focused on the inclusion of a diverse Latinx community, including Spanish speaking and immigrant individuals in Northern California, which gave us tremendous depth in capturing this community’s experiences. However, a limitation of this approach is that our findings may not be generalizable to other Latinx communities. Due to our methodology of door-to-door interviews, our study may be underreporting the prevalence of poor mental health or desire to seek mental health care due to social desirability bias or stigma. However, our partnership with promotores helped to mitigate this by creating a safe space for members of the community to answer the survey. The focus of this study is on the help-seeking experiences and barriers to accessing mental health care, thus we did not collect measures on sustained engagement with mental health treatment or mental health symptoms, and instead relied on self-reporting of mental health concerns. Thus, we do not know to what extent these concerns reflect symptomatology and acknowledge that there are additional barriers to adequate mental health treatment for the Latinx population our study cannot speak to [41]. Lastly, the SHAP used to identify the main barriers for connecting to care is intended to generate hypotheses, with additional findings needed to inform future directions.

## Conclusion

To improve mental health care seeking and connection in the Latinx community, there is a need for culturally tailored interventions to address mental health stigma within the community, to support mental health care navigation, and to increase overall access to care.

## Electronic supplementary material

Below is the link to the electronic supplementary material.

Supplementary Material 1

## Data Availability

Limited aggregate data is available upon reasonable request.

## Declarations

**(Manuscript no.** 76877b99-ea7b-44d3-8846-38563523a40a v1.0**)**
