# Supplementary material for: Mental health care-seeking and barriers: a cross-sectional study of an urban Latinx community
Source: BMC Public Health. 2024 Nov 8;24:3091. doi: 10.1186/s12889-024-20533-6 (PMC11545330; doi:10.1186/s12889-024-20533-6)
Supplement: Supplementary file 1 — Supplementary Material 1 [file 12889_2024_20533_MOESM1_ESM.docx]

**APPENDIX**

**Figure 3. STROBE Diagram**

**
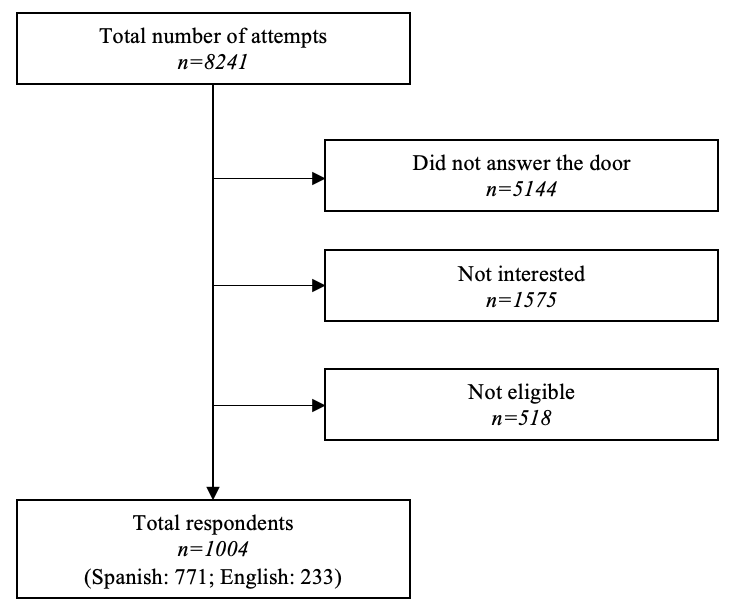
**

**Figure 4. Map of Census Block Groups Sampled in East San José, CA**


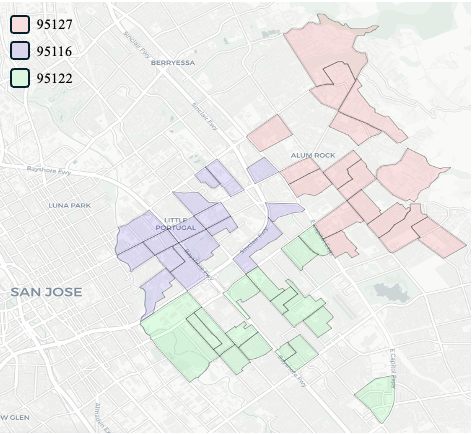


**
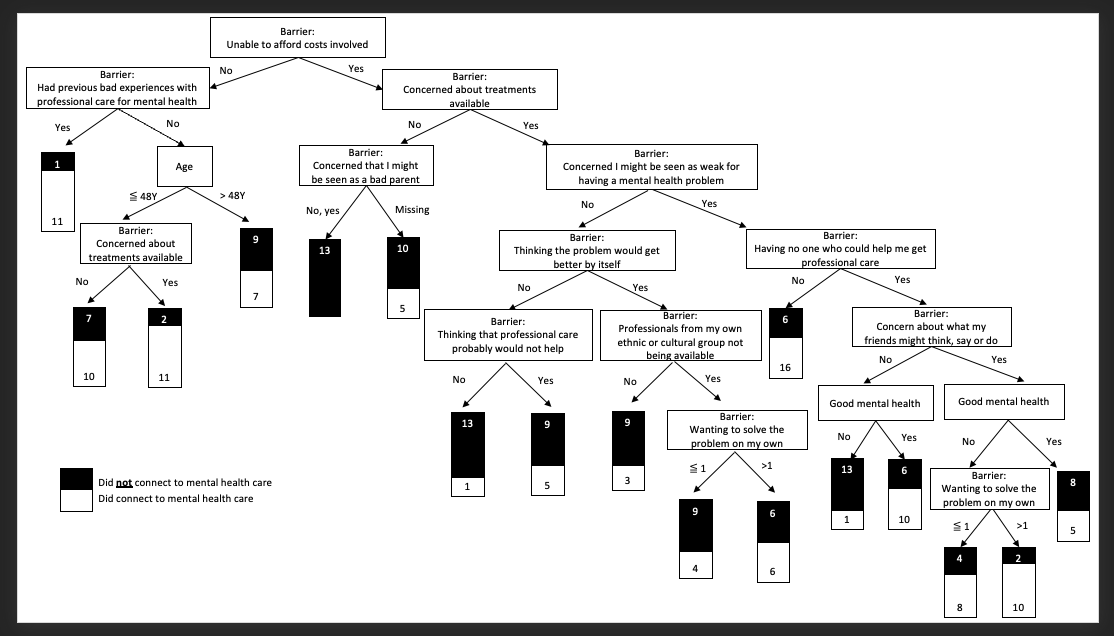
Figure 5. Alternative Decision Tree Obtained from Different Hyperparameters**

**Table 3. Characteristics of Respondents Seeking Lay Provider Mental Health Care**

|  | **Total** N (%) * | **Poor Mental Health** N (%) ** | **Wanted to Connect to Care**  N (%) ** | **Tried to Connect to Care**  N (%) ** | **Connected to Care**  N (%) ** |
| --- | --- | --- | --- | --- | --- |
| **All** | 1004 | 216 (21.5%) | 185 (18.4%) | 136 (13.5%) | 101 (10.1%) |
| **Age group** | | | | | |
| Median (IQR) | 45 (34 - 58) | 48 (31.5 - 59) | 41 (28 – 54) | 42 (31.75 – 55) | 42 (32 – 55) |
| <=25 | 121 (12.1%) | 37/121 (30.6%) | 39/121 (32.2%) | 24/121 (19.8%) | 18/121 (14.9%) |
| 25 - 50 | 495 (49.3%) | 87/495 (17.6%) | 90/495 (18.2%) | 67/495 (13.5%) | 50/495 (10.1%) |
| >50 | 387 (38.5%) | 91/387 (23.5%) | 56/387 (14.5%) | 45/387 (11.6%) | 33/387 (8.5%) |
| Missing | 1 (0.1%) | 1/1 (100.0%) | 0/1 (0.0%) | 0/1 (0.0%) | 0/1 (0.0%) |
| **Gender** | | | | | |
| Male | 355 (35.4%) | 67/355 (18.9%) | 73/355 (20.6%) | 52/355 (14.6%) | 37/355 (10.4%) |
| Female | 638 (63.5%) | 146/638 (22.9%) | 111/638 (17.4%) | 84/638 (13.2%) | 64/638 (10.0%) |
| Prefer not to answer | 6 (0.6%) | 3/6 (50.0%) | 1/6 (16.7%) | 0/6 (0.0%) | 0/6 (0.0%) |
| **Race** | | | | | |
| White | 196 (19.5%) | 42/196 (21.4%) | 30/196 (15.3%) | 22/196 (11.2%) | 17/196 (8.7%) |
| Black or African American | 18 (1.8%) | 3/18 (16.7%) | 4/18 (22.2%) | 3/18 (16.7%) | 3/18 (16.7%) |
| American Indian or Alaskan Native | 60 (6.0%) | 21/60 (35.0%) | 21/60 (35.0%) | 18/60 (30.0%) | 14/60 (23.3%) |
| Asian | 11 (1.1%) | 2/11 (18.2%) | 6/11 (54.5%) | 5/11 (45.5%) | 3/11 (27.3%) |
| Other | 648 (64.5%) | 133/648 (20.5%) | 119/648 (18.4%) | 86/648 (13.3%) | 62/648 (9.6%) |
| Two or more races | 36 (3.6%) | 7/36 (19.4%) | 11/36 (30.6%) | 9/36 (25.0%) | 7/36 (19.4%) |
| **Ethnicity subgroup** | | | | | |
| Mexican, Mexican American, Chicano | 878 (87.5%) | 198/878 (22.6%) | 158/878 (18.0%) | 117/878 (13.3%) | 87/878 (9.9%) |
| Other Latinx or Hispanic | 131 (13.0%) | 16/131 (12.2%) | 29/131 (22.1%) | 20/131 (15.3%) | 15/131 (11.5%) |
| **Insurance type** | | | | | |
| Private | 354 (35.3%) | 64/354 (18.1%) | 71/354 (20.1%) | 54/354 (15.3%) | 40/354 (11.3%) |
| Public | 519 (51.7%) | 122/519 (23.5%) | 95/519 (18.3%) | 68/519 (13.1%) | 52/519 (10.0%) |
| Not covered | 126 (12.5%) | 25/126 (19.8%) | 19/126 (15.1%) | 15/126 (11.9%) | 10/126 (7.9%) |
| **Marital status** | | | | | |
| Married | 454 (45.2%) | 76/454 (16.7%) | 59/454 (13.0%) | 44/454 (9.7%) | 34/454 (7.5%) |
| Divorced/Separated | 129 (12.9%) | 10/129 (7.8%) | 27/129 (20.9%) | 19/129 (14.7%) | 12/129 (9.3%) |
| Widowed | 53 (5.3%) | 14/53 (26.4%) | 10/53 (18.9%) | 8/53 (15.1%) | 6/53 (11.3%) |
| Never married | 208 (20.7%) | 56/208 (26.9%) | 58/208 (27.9%) | 39/208 (18.8%) | 30/208 (14.4%) |
| A member of an unmarried couple | 134 (13.3%) | 32/134 (23.9%) | 27/134 (20.1%) | 22/134 (16.4%) | 16/134 (11.9%) |
| Missing | 26 (2.6%) | 6/26 (23.1%) | 4/26 (15.4%) | 4/26 (15.4%) | 3/26 (11.5%) |
| **Number of children** | | | | | |
| None | 503 (50.1%) | 123/503 (24.5%) | 106/503 (21.1%) | 81/503 (16.1%) | 56/503 (11.1%) |
| Kids in house | 435 (43.3%) | 80/435 (18.4%) | 71/435 (16.3%) | 52/435 (12.0%) | 42/435 (9.7%) |
| Kids out of house | 61 (6.1%) | 12/61 (19.7%) | 7/61 (11.5%) | 2/61 (3.3%) | 2/61 (3.3%) |
| Missing | 5 (0.5%) | 1/5 (20.0%) | 1/5 (20.0%) | 1/5 (20.0%) | 1/5 (20.0%) |
| **Education** | | | | | |
| Never attended | 83 (8.3%) | 27/83 (32.5%) | 10/83 (12.0%) | 7/83 (8.4%) | 5/83 (6.0%) |
| 12th grade or less, no diploma | 370 (36.9%) | 88/370 (23.8%) | 58/370 (15.7%) | 44/370 (11.9%) | 32/370 (8.6%) |
| High school graduate | 295 (29.4%) | 55/295 (18.6%) | 52/295 (17.6%) | 38/295 (12.9%) | 23/295 (7.8%) |
| Some college, no degree | 121 (12.1%) | 25/121 (20.7%) | 27/121 (22.3%) | 19/121 (15.7%) | 17/121 (14.0%) |
| Other degree or certificate | 52 (5.2%) | 10/52 (19.2%) | 14/52 (26.9%) | 9/52 (17.3%) | 7/52 (13.5%) |
| Bachelor or Graduate degree | 77 (7.7%) | 10/77 (13.0%) | 24/77 (31.2%) | 19/77 (24.7%) | 17/77 (22.1%) |
| Missing | 6 (0.6%) | 0/6 (0.0%) | 0/6 (0.0%) | 0/6 (0.0%) | 0/6 (0.0%) |
| **Education in US** | | | | | |
| Completed in US | 415 (41.3%) | 97/415 (23.4%) | 109/415 (26.3%) | 80/415 (19.3%) | 63/415 (15.2%) |
| Not completed in US | 585 (58.3%) | 119/585 (20.3%) | 76/585 (13.0%) | 56/585 (9.6%) | 38/585 (6.5%) |
| Missing | 4 (0.4%) | 0/4 (0.0%) | 0/4 (0.0%) | 0/4 (0.0%) | 0/4 (0.0%) |
| **Nativity** | | | | | |
| Born in US | 296 (29.5%) | 78/296 (26.4%) | 82/296 (27.7%) | 56/296 (18.9%) | 42/296 (14.2%) |
| Foreign born | 704 (70.1%) | 138/704 (19.6%) | 103/704 (14.6%) | 80/704 (11.4%) | 59/704 (8.4%) |
| Missing | 4 (0.4%) | 0/4 (0.0%) | 0/4 (0.0%) | 0/4 (0.0%) | 0/4 (0.0%) |
| **Immigration generation** | | | | | |
| 1st generation | 627 (62.5%) | 125/627 (19.9%) | 82/627 (13.1%) | 61/627 (9.7%) | 42/627 (6.7%) |
| 1.5 generation | 63 (6.3%) | 9/63 (14.3%) | 18/63 (28.6%) | 16/63 (25.4%) | 14/63 (22.2%) |
| Other | 314 (31.3%) | 82/314 (26.1%) | 85/314 (27.1%) | 59/314 (18.8%) | 45/314 (14.3%) |
| **Language preference** | | | | | |
| Spanish | 771 (76.8%) | 157/771 (20.4%) | 116/771 (15.0%) | 91/771 (11.8%) | 66/771 (8.6%) |
| English | 233 (23.2%) | 59/233 (25.3%) | 69/233 (29.6%) | 45/233 (19.3%) | 35/233 (15.0%) |
| **English language proficiency***** | | | | | |
| Very good | 89 (11.5%) | 19/89 (21.3%) | 20/89 (22.5%) | 18/89 (20.2%) | 13/89 (14.6%) |
| Good | 132 (17.1%) | 20/132 (15.2%) | 24/132 (18.2%) | 21/132 (15.9%) | 17/132 (12.9%) |
| Not very well | 390 (50.6%) | 77/390 (19.7%) | 48/390 (12.3%) | 37/390 (9.5%) | 27/390 (6.9%) |
| Not at all | 114 (14.8%) | 34/114 (29.8%) | 19/114 (16.7%) | 12/114 (10.5%) | 6/114 (5.3%) |
| Don’t' know | 29 (8%) | 3/29 (10.3%) | 3/29 (82.8%) | 2/29 (6.9%) | 2/29 (6.9%) |
| Prefer not to answer | 10 (3%) | 2/10 (20.0%) | 1/10 (10.0%) | 1/10 (10.0%) | 1/10 (10.0%) |
| Does not apply | 1 (1%) | 0/1 (0.0%) | 0/1 (0.0%) | 0/1 (0.0%) | 0/1 (0.0%) |
| Missing | 6 (8%) | 2/6 (33.3%) | 1/6 (16.7%) | 0/1 (0.0%) | 0/1 (0.0%) |
| **Frequency of experiencing unfair treatment by people in helping jobs because of the race/ethnic group** | | | | | |
| Never | 736 (73.3%) | 143/736 (19.4%) | 116/736 (15.8%) | 90/736 (12.2%) | 67/736 (9.1%) |
| Once in a while | 122 (12.2%) | 31/122 (25.4%) | 27/122 (22.1%) | 20/122 (16.4%) | 14/122 (11.5%) |
| Sometimes | 105 (10.5%) | 32/105 (30.5%) | 30/105 (28.6%) | 17/105 (16.2%) | 14/105 (13.3%) |
| A lot | 24 (2.4%) | 7/24 (29.2%) | 9/24 (37.5%) | 6/24 (25.0%) | 5/24 (20.8%) |
| Missing | 17 (1.7%) | 3/17 (17.6%) | 3/17 (17.6%) | 3/17 (17.6%) | 1/17 (5.9%) |
| Data presented as N (%), unless otherwise noted  *% in this column are column precents using 1004 as the denominator  **% are row precents using the denominator in the Total column  *** Only asked to participants taking the survey in Spanish | | | | | |
